# A Study on the Glucose Breath Test Positivity Rate and Occurrence of Small Intestine Bacterial Overgrowth-Related Symptoms Caused by Long-Term Use of Proton Pump Inhibitor (PPI) Versus Potassium-Competitive Acid Blocker (P-CAB) in Elderly Patients: SIBO Between PPI and P-CAB

**DOI:** 10.1155/2024/6069151

**Published:** 2024-10-29

**Authors:** Na Rae Lim, Saenal Lim, Woo Chul Chung

**Affiliations:** Department of Internal Medicine, St. Vincent Hospital, The Catholic University of Korea, Seoul, Republic of Korea

**Keywords:** potassium-competitive acid blocker, proton pump inhibitor, small intestinal bacterial overgrowth (SIBO)

## Abstract

**Background/Aims:** Long-term acid suppression with proton pump inhibitors (PPI) leads to hypochlorhydria and facilitates the growth of bacterial flora in the small intestine. Novel acid-suppressants called potassium-competitive acid blockers (P-CABs) seem to be superior to PPIs. However, data on the risk of small intestinal bacterial overgrowth (SIBO) in patients taking P-CABs are limited.

**Method:** We retrospectively analyzed a consecutive series of patients with long-term acid-suppressant (PPIs or P-CABs) use for gastroesophageal reflux disease or nonsteroidal anti-inflammatory drug (NSAID)-induced gastropathy. All of them underwent endoscopic examinations and *Helicobacter pylori* testing and took PPIs or P-CABs for at least 3 months. Glucose hydrogen breath tests (GBT) were performed to check for SIBO, and newly developed SIBO-related symptoms including bloating, postprandial discomfort, diarrheas, and constipation, were evaluated.

**Results:** A total of 142 patients were enrolled. Six patients were excluded due to equivocal *Helicobacter pylori* infection results. The frequency of positive GBTs was 31.7% (25/79) for PPI and 22.8% (13/57) for P-CAB use (*p*=0.15). Regarding GBT positivity, age-related factor was found to be significant in multivariate analysis (*p*=0.02). The results of multivariate analysis in cases of SIBO-related symptoms showed that GBT positivity and PPI use were significant (*p* < 0.01).

**Conclusion:** Long-term use of gastric acid suppressants resulted in positive GBT in approximately 30% of patients, and the risk was particularly high in elderly patients. The occurrence of SIBO-related symptoms was significant in long-term use of PPIs and in patients with positive GBT.

## 1. Introduction

Proton pump inhibitors (PPI) have been widely used to treat patients with acid-related disorders. Many studies have examined the side effects of long-term PPI exposure. Awareness of the side effects of PPIs may be important at this time when drugs with longer half-lives and more powerful suppression of gastric acid are being developed, and the drug use frequency is increasing. In previous studies, the suppression of gastric acid by PPIs was reported to change bacterial composition in the stomach, duodenum, and intestinal tract. Finally, PPI use is a risk factor for small intestinal bacterial overgrowth (SIBO) [1]. Potassium-competitive acid blockers (P-CABs) bind competitively and reversibly to the potassium-binding site of H+/K + ATPase. Compared to conventional PPIs, P-CABs have the following pharmacological advantages. P-CAB is rapidly absorbed into the gastric body, accumulates in parietal cells, and shows a stronger gastric acid inhibitory effect than PPIs from the day of administration.

SIBO occurs when there is an abnormal increase in the overall bacterial population in the small intestine. SIBO causes bloating, diarrhea, or constipation, and related symptoms of nausea, loss of appetite, malnutrition, unintentional weight loss, and abdominal pain. Although there is no single optimal test for diagnosing SIBO, the breath test is a commonly used assessment method in clinical practice. Previously, the glucose hydrogen breath test (GBT) was used to identify SIBO, and the frequency of positive results was significantly higher in the long-term PPI use group compared to the irritable bowel syndrome or control groups [2]. Although previous studies have shown that even short-term PPI use in healthy subjects carries a risk of SIBO [3], an analysis of the duration of PPI use found that SIBO increased with long-term use. The frequency varied depending on the method of diagnosis. When duodenal aspirate fluid was cultured, more frequent SIBO was confirmed in the PPI use group [4]. However, in a prospective study, the association between IBS and SIBO was completely independent of PPI intake, and PPI intake would not prime SIBO [5]. Many studies have suggested a link between SIBO and IBS, but some argue that SIBO might be an epiphenomenon of chronic PPI intake.

Research results on nutrition, metabolism, and infection indicated that long-term PPI use can be relatively safe, but awareness of the side effects is important. It is meaningful to recognize the importance of selecting appropriate indications for PPI use, the minimum dose, and the appropriate duration of PPI use. In this study, we compared the GBT-positive rate and the occurrence of SIBO-related symptoms when PPIs or P-CABs were taken for a long time by high-risk patients with gastroesophageal reflux disease or drug-induced peptic ulcers that required long-term NSAID use.

## 2. Materials and Methods

**Figure 1:**
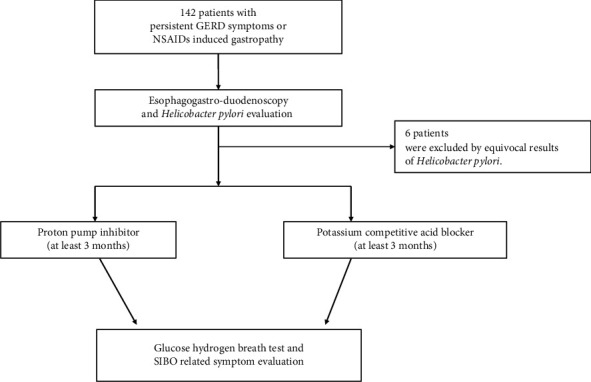
Study flowchart.

This study was approved by the Institutional Review Board of St. Vincent's Hospital, The Catholic University of Korea (approval no. VC22RISI0203). We retrospectively reviewed the charts of patients who were referred to St. Vincent's Hospital, College of Medicine, The Catholic University of Korea for evaluation. We analyzed a consecutive series of patients with long-term acid-suppressant (PPIs or P-CABs; tegoprazan 50 mg, HK Inno N, Seoul, Korea) use due to gastroesophageal reflux disease or NSAID-induced gastropathy. All of them underwent endoscopic examinations and *H*. *pylori* testing and took PPIs or P-CABs for more than 3 months (Figure 1).

*H. pylori* infection status was examined by a rapid urease test (CLO® test; Kimberly-Clark, UT, USA); histological examination, in which we used gastric antral and corpus mucosal tissue; and serum immunoglobulin G (IgG) levels for *H. pylori*. We also performed polymerase chain reaction (PCR) tests. *H. pylori*-negative infections were based on two or more negative results. Cases with confirmed *H. pylori* infections or ambiguous test results were all excluded from the study because SIBO-related symptoms are similar to *H. pylori*-related dyspepsia, making identification ambiguous.

SIBO-related symptoms were defined as abdominal pain, nausea, bloating, constipation, diarrhea, and flatulence [6]. However, abdominal pain was excluded, and the part related to pain was excluded from the analysis, because the symptoms were similar to the purpose of administering GERD drugs. The GBT for SIBO was administered to all patients at the time of administration for more than 3 months, and newly developed SIBO-related symptoms were checked during interviews. At the same time, drugs that could affect the results, such as prokinetics and antibiotics, were taken and excluded from the results analysis. A GBT was performed with the gas chromatography of equipment (SC breathTracker; Quintron Instrument Co., Milwaukee, WI, USA) after an overnight fast of at least 12 h. The patients ingested 50 g of glucose (DIASOL-S SOLN; Taejoon Pharm Co., Ltd., Seoul, Korea), after which duplicate samples of end-expiratory air were collected at baseline and at 10-min intervals for 2 h. The concentrations of breath hydrogen (H₂) and methane (CH₄) were measured using specialized equipment. A baseline hydrogen or methane level greater than 15 parts per million (ppm), or an increase in H_2_ or CH_4_ of ≥ 12 ppm above baseline within 60 min, was considered positive for hydrogen or methane.

## 3. Results

A total of 142 patients were enrolled, and the baseline characteristics are described in Table 1. Patients with equivocal *H. pylori* infection results (*n* = 6) were excluded. The mean ages were 71.83 ± 8.80 and 71.11 ± 9.58 years in the PPI and P-CAB groups, respectively (*p*=0.67). There were 34 (43.0%) and 32 (56.1%) men in the respective groups (*p*=0.13). The patients were followed up for a mean period of 10.66 months (range, 3–60 months). The duration of PPI use was statistically significantly longer than that of P-CAB use (*p*=0.02). There were no significant differences in current smoking, alcohol drinking, the presence of diabetes, or the use of prokinetics between the two groups.

**Figure 2:**
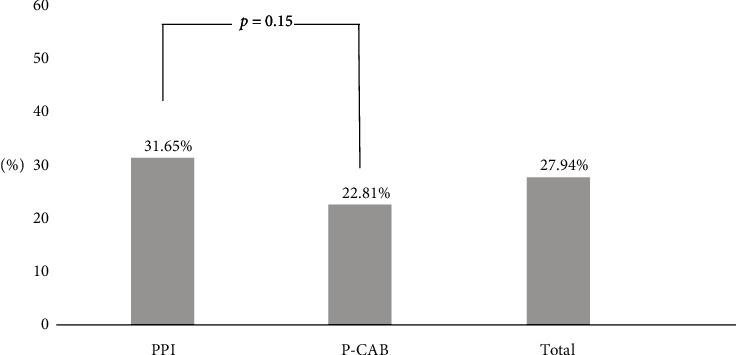
Comparison of positive GBT rates in total, PPI and P-CAB use groups. PPI = proton pump inhibitor, P-CAB = potassium competitive acid blocker.

The frequency of positive GBTs was 31.7% (25/79) for PPI and 22.8% (13/57) for P-CAB use (*p*=0.15) (Figure 2). Most of the cases showed dominant breath hydrogen positivity, while combined hydrogen and methane positivity was observed in 1 out of 25 patients in the PPI group and 1 out of 13 patients in the P-CAB group. There were no cases of dominant methane positivity. In multivariable regression analysis (Table 2), age was significantly associated with positive GBTs (95% confidence interval (CI): 1.008–1.117, *p*=0.02) (Table 2). Determining the presence or absence of SIBO using the GBT reflects the state of the upper jejunum to some extent, but it is difficult to know the state of the lower ileum. Therefore, the present research team investigated the presence or absence of newly occurring SIBO-related symptoms (abdominal pain, nausea, bloating, constipation, diarrhea, and flatulence) after using the drug as an additional factor.

**Figure 3:**
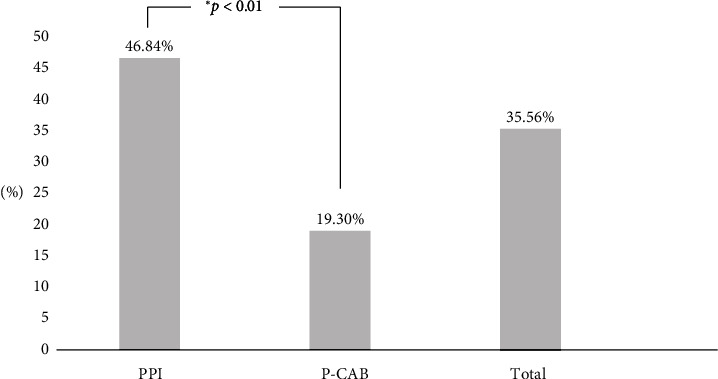
Comparison of the presence of SIBO-related symptoms in total, PPI, and P-CAB use groups. PPI = proton pump inhibitor, P-CAB = potassium competitive acid blocker.

The frequency of the presence of SIBO related symptoms was 46.8% (37/79) for PPI and 19.3% (11/57) for P-CAB use (*p* < 0.01) (Figure 3). In multivariable regression analysis (Table 3), PPI use (95% CI: 1.519–10.040, *p* < 0.01), and GBT positive results (95% CI: 3.279–21.277, *p* < 0.01) were significant correlated.

## 4. Discussion

It is known that SIBO is diagnosed at a lower frequency than when actually investigated, because SIBO may be asymptomatic or nonspecific. An accurate method of diagnosing SIBO is to identify and quantify microorganisms through aspiration microbiological analysis. In early studies of SIBO, a diagnosis was made when the number exceeded 10^5^ colony-forming units (CFU)/mL [7]. These bacteria are usually coliforms, which are typically found in the colon and include predominantly Gram-negative aerobic and anaerobic species that ferment carbohydrates, producing gas [8]. However, there were issues to be addressed. The validity of this criterion itself was not verified, and the aspiration method was diverse, including different aspiration locations, aspiration amounts, and culture methods [9]. It was adequately diagnosed in only a small number of cases, and the prevalence of SIBO varied according to the disease. The diagnosis rate was 14–85% in patients with irritable bowel syndrome and up to 50% in patients with celiac disease who did not respond to a gluten-restricted diet [10].

The breath test is an indirect method to determine SIBO and is currently replacing aspiration microbiological analysis in clinical practice. The SIBO breath test measures hydrogen or methane in an exhaled breath sample. Carbohydrate substrates, such as lactulose or glucose, are metabolized by microorganisms in the small intestine to hydrogen or methane, which are then exhaled. Lactulose, the substrate used in the breath test, is not normally absorbed in the small intestine. Thus, patients with SIBO may show maxima in the initial sample of exhaled hydrogen or methane due to lactulose metabolism by abnormal small intestinal flora. However, false-positive results may occur when using lactulose as a substrate if the patient has a rapid colonic transit time. Glucose is used as a substrate for the breath test to compensate for these disadvantages. Glucose is normally absorbed in the proximal small intestine and metabolized by bacteria to hydrogen. Therefore, hydrogen levels in exhaled samples are elevated in patients with SIBO. However, there is no current gold standard diagnostic test for SIBO. Both small bowel aspiration and breath tests have limitations as diagnostic tests. Therefore, diagnosis is based on the identification of symptoms, medical history, and risk factors, and excluding other similar conditions. In this study, to determine the presence of SIBO in patients receiving long-term gastric acid suppression therapy, we did not simply rely on blood glucose breath test results, but simultaneously investigated new SIBO-related symptoms. However, the occurrence of SIBO-related symptoms does not directly prove SIBO, but it is expected to be directly or indirectly related in many ways.

SIBO might be related to the presence of gastrointestinal cancer. However, the exact link between SIBO and cancer prevalence, as well as cancer symptoms, remains unclear. The SIBO-positive rate in patients with esophageal cancer, gastric cancer, and liver cancer were 47.1% (16/34), 49.4% (41/83), and 76.5% (39/51), respectively [11], suggesting that patients with digestive disease are prone to SIBO. *Escherichia coli* (*E. coli*), which is the most abundant strain in the intestine, are also closely related to the growth of colorectal cancer. A previous study reported that the level of mucosal-associated *E. coli* was increased in colorectal cancer tissue compared to normal colon tissues [12]. In addition, PPIs increased the risk of gastric neuroendocrine tumors, increased gastric adenocarcinoma in association with *H. pylori* infection, and induced carcinogenic progression in a subset of patients with a specific predisposition to Barrett's esophagus [13]. Thus, there are case reports of PPI-induced gastric NETs and adenocarcinoma [14, 15]. The mechanism proposed for this process is that PPI-induced hypo- or achlorhydria may lead to the overgrowth of other orally ingested bacteria. As a result, nitrosamine production is increased and *H. pylori* and non-*H. pylori* are thought to be involved in the formation of malignant neoplasms through interactions [16, 17]. Future studies should also confirm the clinical effects related to hypergastrinemia and bacterial overgrowth from long-term PPI use.

Because P-CABs have a long duration of action, they maintain pH values above four in the stomach longer than PPIs. In addition, P-CABs do not require acid activation and can act faster. Therefore, P-CABs have no choice but to worry about the same level as PPIs in terms of other complications that may occur in connection with long-term use. Of note, regarding the occurrence of SIBO, P-CABs have the unique effect of stimulating migration motor complex (MMC) Phase III [18]. MMC is a cyclic, recurring motility pattern that occurs in the stomach and small bowel during fasting. Phase III of the MMC is the most active and digestive juice secretion is also increased. Intestinal movement and digestive juice secretion are physiologically effective. In particular, Phase III of the MMC clears the small bowel of debris [19] and is also known as the housekeeper. The physiological role of the MMC is incompletely understood, but it is known to play some protective role against SIBO by removing food debris and bacteria left during peristalsis and segment contraction in the small intestine [20, 21]. This effect is similar to adding prokinetics, which are used to partially prevent SIBO caused by long-term PPI use. A previous study reported that the use of prokinetics by patients taking PPIs could reduce the risk of SIBO by enhancing intestinal motility [22]. In the present study, there was no statistically significant difference in GBT results between the PPI and the P-CAB group, but the presence of SIBO-related symptoms was significantly lower in the P-CAB group. The higher prevalence of GBT positivity and SIBO symptoms might be due to patients taking PPIs for a significantly longer duration compared to P-CABs, though no significant difference of duration was found in the multivariate analysis. Additional follow-up studies are needed to confirm whether long-term P-CAB use leads to SIBO. There are limitations to this study: it was a retrospective pilot study, and the percentage of patients with pre-existing SIBO before taking the test drugs was unknown. Therefore, in-depth research with a larger patient population should be conducted to further clarify these results.

In conclusion, approximately 30% of patients show GBT positivity due to long-term gastric acid suppression treatment, and the risk increases in elderly patients. P-CAB users showed a significant prevalence of SIBO, though not as high as PPI users. Moreover, the occurrence of SIBO-related symptoms occurs in a significant number of cases, and the frequency is particularly significant in cases of PPI use and in GBT-positive patients. Therefore, elderly patients taking PPIs for a long period of time should pay attention to the risk of developing SIBO.

## Figures and Tables

**Table 1:** Baseline characteristics.

Variable	PPI user (*n* = 79)	P-CAB user (*n* = 57)	*p* value
Age	71.83 ± 8.80	71.11 ± 9.58	0.67
Male sex	34	32	0.13
Current smoker	10	9	0.60
Alcohol drinking	17	8	0.27
Diabetes mellitus	18	10	0.46
Duration of drug use (months)	12.93 ± 14.16	7.43 ± 6.38	0.02⁣^∗^
Underlying disease			
(GERD)	39	21	0.15
(Gastric protection for NSAIDs)	40	36	
Use of prokinetics	27	17	0.59

Abbreviations: GERD = gastroesophageal reflux disease, PPI = proton pump inhibitor, P-CAB = potassium competitive acid blocker.

⁣^∗^Statistically significant.

**Table 2:** Multivariate analysis for the results of glucose hydrogen breath test (GBT).

Variable	Odds ratio (95% CI)	*p* value
Age	1.061 (1.008–1.117)	0.02⁣^∗^
Male sex	0.574 (0.230–1.432)	0.23
Current smoker	0.564 (0.163–1.951)	0.36
Alcohol	0.567 (0.224–1.438)	0.12
Diabetes mellitus	1.058 (0.424–2.638)	0.79
Use of proton pump inhibitor (PPI)	1.342 (0.573–3.143)	0.48
Duration of drug use	1.017 (0.984–1.051)	0.31
Underlying disease (GERD/gastric protection of NSAIDs)	0.634 (0.273–1.473)	0.28
Use of prokinetics	0.974 (0.408–2.323)	0.95

Abbreviation: GERD = gastroesophageal reflux disease.

⁣^∗^Statistically significant.

**Table 3:** Multivariate analysis for the results of the presence of small bowel bacterial overgrowth-related symptoms.

Variable	Odds ratio (95% CI)	*p*-value
Age	1.021 (0.971–1.072)	0.42
Male sex	1.158 (0.446–3.003)	0.76
Current smoker	1.269 (0.360–4.464)	0.71
Alcohol	1.380 (0.469–4.057)	0.56
Diabetes mellitus	1.039 (0.357–3.020)	0.94
Use of proton pump inhibitor (PPI)	3.906 (1.519–10.040)	<0.01⁣^∗^
Duration of drug use	0.995 (0.959–1.033)	0.63
Underlying disease (GERD/gastric protection of NSAIDs)	1.242 (0.520–2.964)	0.48
Use of prokinetics	0.748 (0.301–1.859)	0.75
Positive results of GBT	8.333 (3.279–21.277)	<0.01⁣^∗^

Abbreviations: GERD = gastroesophageal reflux disease, GBT = glucose breath test, NSAIDs = nonsteroidal anti-inflammatory drugs.

⁣^∗^Statistically significant.

## Data Availability

The data that support the findings of this study are openly available.

## References

[1] Williams C., McColl K. E. (2006). Review Article: Proton Pump Inhibitors and Bacterial Overgrowth. *Alimentary Pharmacology & Therapeutics*.

[2] Lombardo L., Foti M., Ruggia O., Chiecchio A. (2010). Increased Incidence of Small Intestinal Bacterial Overgrowth During Proton Pump Inhibitor Therapy. *Clinical Gastroenterology and Hepatology*.

[3] Duran-Rosas C., Priego-Parra B. A., Morel-Cerda E. (2024). Incidence of Small Intestinal Bacterial Overgrowth and Symptoms After 7 Days of Proton Pump Inhibitor Use: A Study on Healthy Volunteers. *Digestive Diseases and Sciences*.

[4] Thorens J., Froehlich F., Schwizer W. (1996). Bacterial Overgrowth During Treatment With Omeprazole Compared with Cimetidine: A Prospective Randomised Double Blind Study. *Gut*.

[5] Giamarellos-Bourboulis E. J., Pyleris E., Barbatzas C., Pistiki A., Pimentel M. (2016). Small Intestinal Bacterial Overgrowth Is Associated With Irritable Bowel Syndrome and Is Independent of Proton Pump Inhibitor Usage. *BMC Gastroenterology*.

[6] Compare D., Pica L., Rocco A. (2011). Effects of Long-Term PPI Treatment on Producing Bowel Symptoms and SIBO. *European Journal of Clinical Investigation*.

[7] Bushyhead D., Quigley E. M. (2021). Small Intestinal Bacterial Overgrowth. *Gastroenterology Clinics of North America*.

[8] Pimentel M., Saad R. J., Long M. D., Rao S. S. C. (2020). ACG Clinical Guideline: Small Intestinal Bacterial Overgrowth. *American Journal of Gastroenterology*.

[9] Khoshini R., Dai S. C., Lezcano S., Pimentel M. (2008). A Systematic Review of Diagnostic Tests for Small Intestinal Bacterial Overgrowth. *Digestive Diseases and Sciences*.

[10] Bures J., Cyrany J., Kohoutova D. (2010). Small Intestinal Bacterial Overgrowth Syndrome. *World Journal of Gastroenterology*.

[11] Liang S., Xu L., Zhang D., Wu Z. (2016). Effect of Probiotics on Small Intestinal Bacterial Overgrowth in Patients With Gastric and Colorectal Cancer. *Turkish Journal of Gastroenterology*.

[12] Bonnet M., Buc E., Sauvanet P. (2014). Colonization of the Human Gut by *E. coli* and Colorectal Cancer Risk. *Clinical Cancer Research*.

[13] Ko Y., Tang J., Sanagapalli S., Kim B. S., Leong R. W. (2016). Safety of Proton Pump Inhibitors and Risk of Gastric Cancers: Review of Literature and Pathophysiological Mechanisms. *Expert Opinion on Drug Safety*.

[14] Jianu C. S., Lange O. J., Viset T. (2012). Gastric Neuroendocrine Carcinoma after Long-Term Use of Proton Pump Inhibitor. *Scandinavian Journal of Gastroenterology*.

[15] Waldum H. L., Rehfeld J. F. (2019). Gastric Cancer and Gastrin: On the Interaction of *Helicobacter pylori* Gastritis and Acid Inhibitory Induced Hypergastrinemia. *Scandinavian Journal of Gastroenterology*.

[16] Sanduleanu S., Jonkers D., De Bruine A., Hameeteman W., Stockbrugger R. W. (2001). Double Gastric Infection With *Helicobacter pylori* and Non-Helicobacter pylori Bacteria During Acid-Suppressive Therapy: Increase of Pro-Inflammatory Cytokines and Development of Atrophic Gastritis. *Alimentary Pharmacology & Therapeutics*.

[17] Sugano K. (2013). Premalignant Conditions of Gastric Cancer. *Journal of Gastroenterology and Hepatology*.

[18] Takahashi N., Take Y. (2018). Tegoprazan, A Novel Potassium-Competitive Acid Blocker to Control Gastric Acid Secretion and Motility. *Journal of Pharmacology and Experimental Therapeutics*.

[19] Vantrappen G., Janssens J., Hellemans J., Ghoos Y. (1977). The Interdigestive Motor Complex of Normal Subjects and Patients With Bacterial Overgrowth of the Small Intestine. *Journal of Clinical Investigation*.

[20] Deloose E., Janssen P., Depoortere I., Tack J. (2012). The Migrating Motor Complex: Control Mechanisms and Its Role in Health and Disease. *Nature Reviews Gastroenterology & Hepatology*.

[21] Deloose E., Tack J. (2016). Redefining the Functional Roles of the Gastrointestinal Migrating Motor Complex and Motilin in Small Bacterial Overgrowth and Hunger Signaling. *American Journal of Physiology-Gastrointestinal and Liver Physiology*.

[22] Revaiah P. C., Kochhar R., Rana S. V. (2018). Risk of Small Intestinal Bacterial Overgrowth in Patients Receiving Proton Pump Inhibitors Versus Proton Pump Inhibitors Plus Prokinetics. *JGH Open*.

